# Estimate of Illnesses from *Salmonella* Enteritidis in Eggs, United States, 2000

**DOI:** 10.3201/eid1101.040401

**Published:** 2005-01

**Authors:** Carl M. Schroeder, Alecia Larew Naugle, Wayne D. Schlosser, Allan T. Hogue, Frederick J. Angulo, Jonathon S. Rose, Eric D. Ebel, W. Terry Disney, Kristin G. Holt, David P. Goldman

**Affiliations:** * U.S. Food Safety and Inspection Service, Washington, DC, USA; † U. S. Food Safety and Inspection Service, College Station, Texas, USA; ‡ U.S. Animal and Plant Health Inspection Service, Riverdale, Maryland, USA; § Centers for Disease Control and Prevention, Atlanta, Georgia, USA; ¶ U.S. Animal and Plant Health Inspection Service, Ft. Collins, Colorado, USA; # U.S. Food Safety and Inspection Service, Fort. Collins, Colorado, USA

**Keywords:** *Salmonella* Enteritidis, salmonellosis, eggs, estimates, illnesses, uncertainty, dispatch

## Abstract

Results from our model suggest that eating *Salmonella enterica* serovar Enteritidis–contaminated shell eggs caused 182,060 illnesses in the United States during 2000. Uncertainty about the estimate ranged from 81,535 (5th percentile) to 276,500 illnesses (95th percentile). Our model provides 1 approach for estimating foodborne illness and quantifying estimate uncertainty.

Foodborne salmonellae are estimated to cause ≈1.3 million illnesses, 15,000 hospitalizations, and 500 deaths per year in the United States (*1*). *Salmonella enterica* serovar Enteritidis is a leading cause of foodborne salmonellosis. After its emergence in the northeastern United States during the late 1970s, the *S.* Enteritidis epidemic spread throughout the country. It was detected in the Atlantic region in 1984 and the Pacific region in 1993 (*2*,*3*). Culture-confirmed *S.* Enteritidis infections peaked at ≈4/100,000 population in 1995 and declined to ≈2/100,000 in 1999 (*4*). Eggs and egg-containing foods are the primary vehicles of *S.* Enteritidis infection, having been implicated in 298 (80%) of the 371 known-source *S.* Enteritidis outbreaks reported to the Centers for Disease Control and Prevention (CDC) from 1985 through 1999 (*4*). Nevertheless, the annual number of shell egg–associated *S.* Enteritidis illnesses in the United States is unknown. One estimate suggested that 200,000 to 1 million *S.* Enteritidis illnesses occurred in the United States in 1996 (*2*), but it was not specific for those attributed to shell egg consumption. Using data from the Foodborne Diseases Active Surveillance Network (FoodNet), we developed a model to estimate the number of shell egg–associated *S.* Enteritidis illnesses in the United States for 2000. The model was also designed to quantify estimate uncertainty.

## The Study

The estimated number of illnesses from shell egg–associated *S.* Enteritidis in the United States for the year 2000 (Ill_SE_) was calculated as: 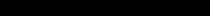

(equation 1), where

Ill_SE_ = number of *S.* Enteritidis illnesses from eating shell eggs in 2000.

F1 = number of culture-confirmed salmonellosis cases ascertained by FoodNet in 2000 = 4,330.

F2 = the proportion of culture-confirmed salmonellosis cases ascertained by FoodNet for which isolates were serotyped as *S.* Enteritidis. From the 4,330 culture-confirmed salmonellosis cases ascertained for 2000, 3,964 *Salmonella* isolates were serotyped, 585 of which were identified as *S.* Enteritidis (585/3,964 = 0.148).

F3 = the proportion of *S.* Enteritidis cases from eating shell eggs. In 2000, FoodNet ascertained 15 *S.* Enteritidis outbreaks in which food vehicles were identified: 12 were egg-associated (12/15 = 0.8). This proportion was used as a surrogate for the proportion of sporadic *S.* Enteritidis illnesses from eating shell eggs.

F4 = a multiplier to account for cases of salmonellosis that occurred in the FoodNet catchment area but were not confirmed by fecal culture, and subsequently, not ascertained by FoodNet. The value used for this multiplier was 38.6 (*5*).

F5 = a multiplier to extrapolate from the FoodNet catchment area to the U.S. population. For 2000, the population in the 8 FoodNet catchment sites was 30,500,000 persons, thus representing 10.8% of the U.S. population at that time (*6*). The multiplier was computed by taking the inverse of the proportion of the U.S. population represented by the catchment area (1/0.108 = 9.2).

Thus, based on equation 1 above, the Ill_SE_ point estimate was calculated as: 4,330 x (585/3,964) x (12/15) x 38.6 x (281,400,000/30,500,000) = 182,060.

Uncertainty for the estimate of Ill_SE_ was also determined. As illustrated in equation 1, multipliers F2, F3, F4, and F5 adjusted the number of culture-confirmed salmonella illnesses ascertained by FoodNet in 2000 (F1) to estimate the number of *S.* Enteritidis illnesses due to eating shell eggs. Uncertainty associated with each multiplier contributes to the overall uncertainty associated with the estimate of Ill_SE_. The distributions described below were incorporated into a Monte Carlo simulation (@RISK, version 4.0, Palisade Corp., Newfield, NY) of 100,000 iterations to estimate the range of potential values for Ill_SE_ (Figure).

**Figure:**
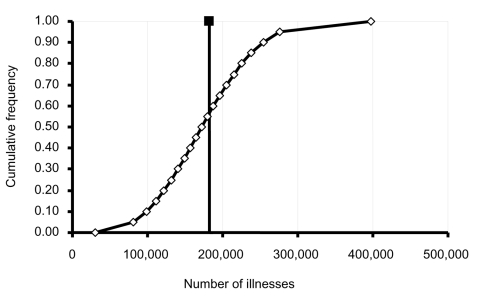
Estimated number of illnesses from *Salmonella* Enteritidis in shell eggs, United States, 2000. The point estimate of 182,060 illnesses is indicated by the filled box and solid vertical line. The open diamonds and attached line indicate the range of estimate uncertainty (5th percentile = 81,535 illnesses, 95th percentile = 276,500 illnesses).

F2 assumed the proportion of *Salmonella* illnesses attributable to *S.* Enteritidis ascertained by FoodNet was equal to the proportion of *Salmonella* illnesses attributable to *S.* Enteritidis throughout the United States. The β distribution (585 + 1, 3,964 – 585 + 1) was used to describe uncertainty around the F2 point value.

F3 assumed that the proportion of *S.* Enteritidis outbreaks and sporadic infections attributable to eating shell eggs was equivalent. The β distribution, (12 + 1, 15 – 12 + 1), was used to model the uncertainty around the proportion of *S.* Enteritidis cases assumed to have resulted from shell egg consumption.

F4 assumed that the impact of diarrheal illness, and the behavior of persons with diarrhea and their healthcare providers, was the same in the FoodNet catchment area as in the U.S. population. It also assumed that the proportions of case-patients who 1) sought medical attention, 2) provided a specimen for fecal culture, and 3) were confirmed as salmonellosis patients contributed equally to case ascertainment, but that these proportions differed for patients who experienced bloody diarrhea compared to those who experienced nonbloody diarrhea. A triangular distribution with a minimum value of 9.8 and a maximum value of 67.7 around the point estimate of 38.6 was specified to quantify uncertainty associated with F4.

F5 assumed that the population of the FoodNet catchment area in 2000 was representative of the U.S. population. Because this assumption was qualitative, uncertainty associated with the multiplier could not be modeled.

## Conclusions

We estimated that 182,060 illnesses due to egg-associated *S.* Enteritidis occurred during 2000 (Figure). Based on previous estimates that suggested that the ratio of illnesses to hospitalizations to deaths for nontyphoidal salmonellosis is roughly 2,426 to 28 to 1 (*1*), our estimate extrapolates to ≈2,000 hospitalizations and 70 deaths. In recognition of the fact that descriptions of the impact of illness from foodborne pathogens are inexact, our model was designed to characterize uncertainty about the estimate of illnesses resulting from eggborne *S.* Enteritidis. Ninety percent of the model iterations resulted in estimates of the number of shell egg–associated *S.* Enteritidis illnesses from 81,535 (5th percentile) to 276,500 (95th percentile) (Figure). Because the proportion of *S.* Enteritidis illnesses attributed to eggs was based on a relatively small number of outbreaks (15 outbreaks), the uncertainty about this multiplier was an important contributor to the overall uncertainty in our estimate. Angulo and Swerdlow (*2*) estimated that 200,000 to 1 million *S.* Enteritidis infections occurred in the United States in 1996. The lower range of our estimate in part reflects that it was computed for only those *S.* Enteritidis infections from eggs and that the relative number of *S.* Enteritidis infections reported by FoodNet was lower in 2000 than in 1996 (*7*).

Several assumptions were made in this study. First, all culture-confirmed salmonellosis cases in the FoodNet catchment area were assumed to have been ascertained through FoodNet, a reasonable assumption considering FoodNet is an active surveillance system. Second, the proportion of *Salmonella* isolates identified as *S.* Enteritidis in FoodNet sites was assumed to be comparable to that identified nationally. The proportion derived from FoodNet (≈15%) was similar to that reported for 2000 through the Public Health Laboratory Information System (PHLIS) (≈19%) (*8*). Third, the proportion of *S.* Enteritidis cases from shell eggs was assumed to be similar between the FoodNet catchment area and the nation. The value derived from FoodNet (80%) for 2000 was identical to that for 1985 through 1999, as reported through CDC’s National *Salmonella* Surveillance System (*4*). Fourth, the multiplier for underascertainment was assumed to be correct. Granted, FoodNet data are limited to diagnosed illnesses, whereas most foodborne illnesses are neither diagnosed nor reported: nevertheless, the value of 38.6 used here was derived specifically for the estimation of salmonellosis cases from FoodNet data (*5*). Lastly, the population of the FoodNet catchment area in 2000 was assumed to be representative of the U.S. population, although FoodNet findings may not be generalizable to the nation.

Findings of this study suggest eggborne *S.* Enteritidis was an important public health problem in the United States during 2000. The findings also illustrate the potential for uncertainty in estimating the impact of foodborne illness. The model we described here provides but one approach for estimating foodborne illness and quantifying estimate uncertainty.
